# Yiqi Jiemin decoction alleviates allergic rhinitis in a guinea pig model by suppressing inflammation, restoring Th1/Th2 balance, and improving cellular metabolism

**DOI:** 10.18632/aging.203292

**Published:** 2021-07-27

**Authors:** Zhanfeng Yan, Lili Liu, Jingjing Yuan, Lulu Jiao, Mo Zhou, Jinfeng Liu, Xiaohui Wen, Siming Liu, Pengpeng Hao, Jianhua Liu, Wei Wu

**Affiliations:** 1 Department of Otorhinolaryngology, Dongzhimen Hospital, The First Affiliated Hospital of Beijing University of Chinese Medicine, Beijing 100000, China; 2 Department of Otorhinolaryngology Head and Neck Surgery, Beijing Chaoyang Hospital, Capital Medical University, Beijing 100000, China

**Keywords:** allergic rhinitis, Yiqi Jiemin decoction

## Abstract

We investigated the mechanisms underlying the therapeutic effects of Yiqi Jiemin decoction (YJD), a traditional Chinese medicine (TCM), in the ovalbumin (OVA)-induced allergic rhinitis (AR) model in guinea pigs. YJD significantly decreased infiltration of mast cells and eosinophils into the nasal mucosa of AR model guinea pigs. YJD also increased expression of TGF-β in the nasal mucosa, restored the balance of Th1/Th2 immune cell responses, and decreased serum levels of various pro-inflammatory mediators, including histamine (HA), neuropeptide Y (NPY), acetylcholine (ACH), norepinephrine and immunoglobulin E (IgE). Metabolic analyses using liquid chromatography coupled with high-resolution mass spectrometry revealed that YJD improved cellular metabolism in AR model guinea pigs and increased serum levels of glycocholic acid while decreasing levels 1-palmitoyl lysophosphatidic acid. RNA-sequencing analysis identified *BPIFB2* as a potential diagnostic biomarker and therapeutic target for AR. Functional enrichment analyses showed that YJD significantly inhibited cytokine secretion pathways in AR model guinea pigs. These findings demonstrate that YJD protects against OVA-induced AR in guinea pigs by suppressing inflammation in the nasal mucosa, restoring Th1/Th2 balance, and improving cellular metabolism.

## INTRODUCTION

Allergic rhinitis (AR) is an IgE-mediated inflammatory disease of the nasal mucosa in response to allergen exposure, with symptoms such as airflow obstruction, nasal itching, rhinorrhoea, and sneezing [1]. The worldwide incidence of AR has increased dramatically over the last few decades [2, 3]. The prevalence of allergic rhinitis is about 10%-30% in adults and approximately 40% in children [4]. AR significantly impacts the quality of life including school activities of pediatric patients and job productivity of adult patients.

Th1/Th2 cytokine imbalance is a key feature of AR [5]. During allergen exposure, peripheral blood mononuclear cells of AR patients secrete aberrantly high levels of Th2 cytokines and chemokines [6, 7]. These promote excessive infiltration of eosinophils (EOS) and mast cells (MC) that release large amounts of leukotrienes, histamine, and other pro-inflammatory factors in the nasal mucosa, thereby inducing local inflammation, and mucosal damage [8–10]. AR is clinically diagnosed by thorough evaluation of patient medical history as well as allergen testing with *in vivo* skin prick tests (SPTs) and *in vitro* estimation of serum allergen-specific IgE levels [11–13]. Second-generation H1 anti-histamines, nasal glucocorticosteroids, and leukotriene antagonists are the first-line drugs for AR patients, whereas, allergen immunotherapy and surgery is prescribed when the first-line drugs are ineffective [2, 14]. In most cases, drugs provide only temporary relief and symptoms relapse after drug withdrawal [15]. Therefore, there is an urgent need to identify effective therapeutic strategies for AR patients.

Traditional Chinese medicine (TCM) has been widely accepted and practiced in China for more than two thousand years [16, 17]. Previous studies have shown that acupoint herbal patching alone or in combination with western medicine is more effective in AR patients than treatment with placebo or western medicine alone [18, 19]. Yan et al. found that intranasal acupuncture combined with Yiqi Jiemin Decoction significantly attenuated the symptom of moderate-severe allergic rhinitis with deficiency of lung and spleen qi [20]. However, the underlying mechanism of Yiqi Jiemin Decoction (YJD), another commonly used TCM for AR, is not known. Therefore, in this study, we performed integrated metabolomics and transcriptomics analyses to determine the underlying therapeutic mechanisms of YJD in the AR model of guinea pigs.

## RESULTS

### YJD treatment alleviates AR symptoms and nasal mucosa injury in the AR model guinea pigs

**Figure 1:**
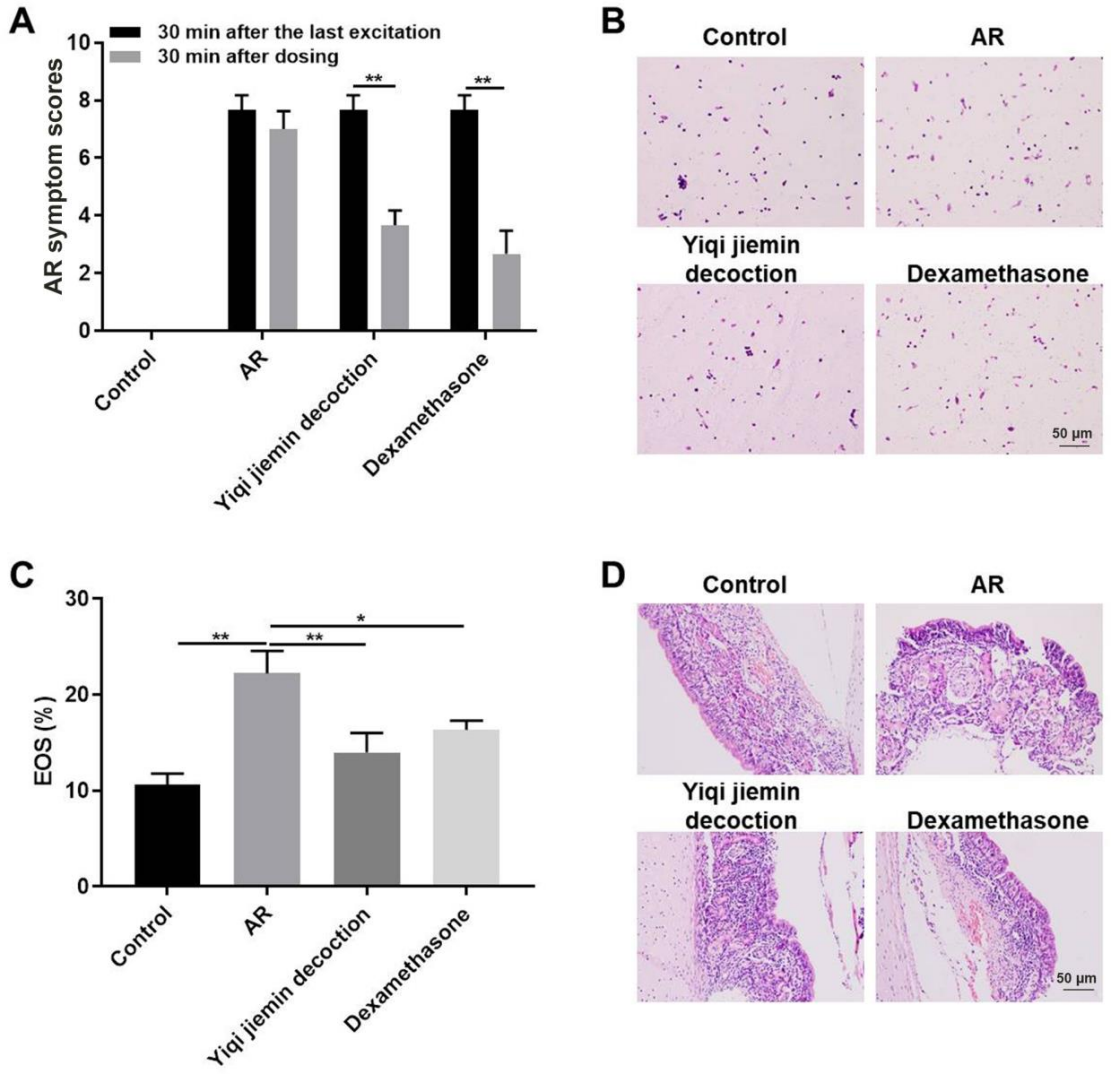
**YJD treatment alleviates nasal symptoms in AR model guinea pigs.** (**A**) The histogram plots show AR symptom scores for the control, AR model, AR model plus YJD, and AR model plus dexamethasone groups of guinea pigs. The data are represented as means ± SD from 3 independent experiments. *P < 0.05; **P < 0.01; two-way ANOVA. (**B**) Wright’s-Giemsa staining results show histological changes in nasal mucosal samples from the control, AR model, AR model plus YJD, and AR model plus dexamethasone groups. (**C**) The histogram plots show the percentage of eosinophils in the Wright’s-Giemsa-stained samples of nasal mucosal samples from the control, AR model, AR model plus YJD, and AR model plus dexamethasone groups. The data was independently analyzed by 3 pathologists based on five different views under a light microscope. *P < 0.05; **P < 0.01; one-way ANOVA. (**D**) Representative H&E-stained images of nasal mucosal samples from the control, AR model, AR model plus YJD, and AR model plus dexamethasone groups.

We used the clinical nasal symptom scoring system to determine the effects of YJD on the AR model guinea pigs. In addition, dexamethasone is a glucocorticoid steroid used to treat many diseases including allergic rhinitis, which used as a positive control [21, 22]. The pathological scores were significantly higher for the AR model group compared to the control group; but were significantly reduced for the AR model guinea pigs treated with YJD or dexamethasone (Figure 1A). Wright-Giemsa staining results showed massive infiltration of eosinophils in the nasal mucosa in the AR model group guinea pigs, but, these effects were significantly reduced by YJD and dexamethasone treatments (Figure 1B, 1C). H&E staining results showed that YJD and dexamethasone treatments reduced OVA-induced mucosal injury and sub-mucosal inflammatory edema in the AR model guinea pigs (Figure 1D). These results demonstrated that YJD significantly reduced OVA-induced AR in the guinea pig model.

### YJD treatment inhibits OVA-induced inflammation in the AR model guinea pigs

**Figure 2:**
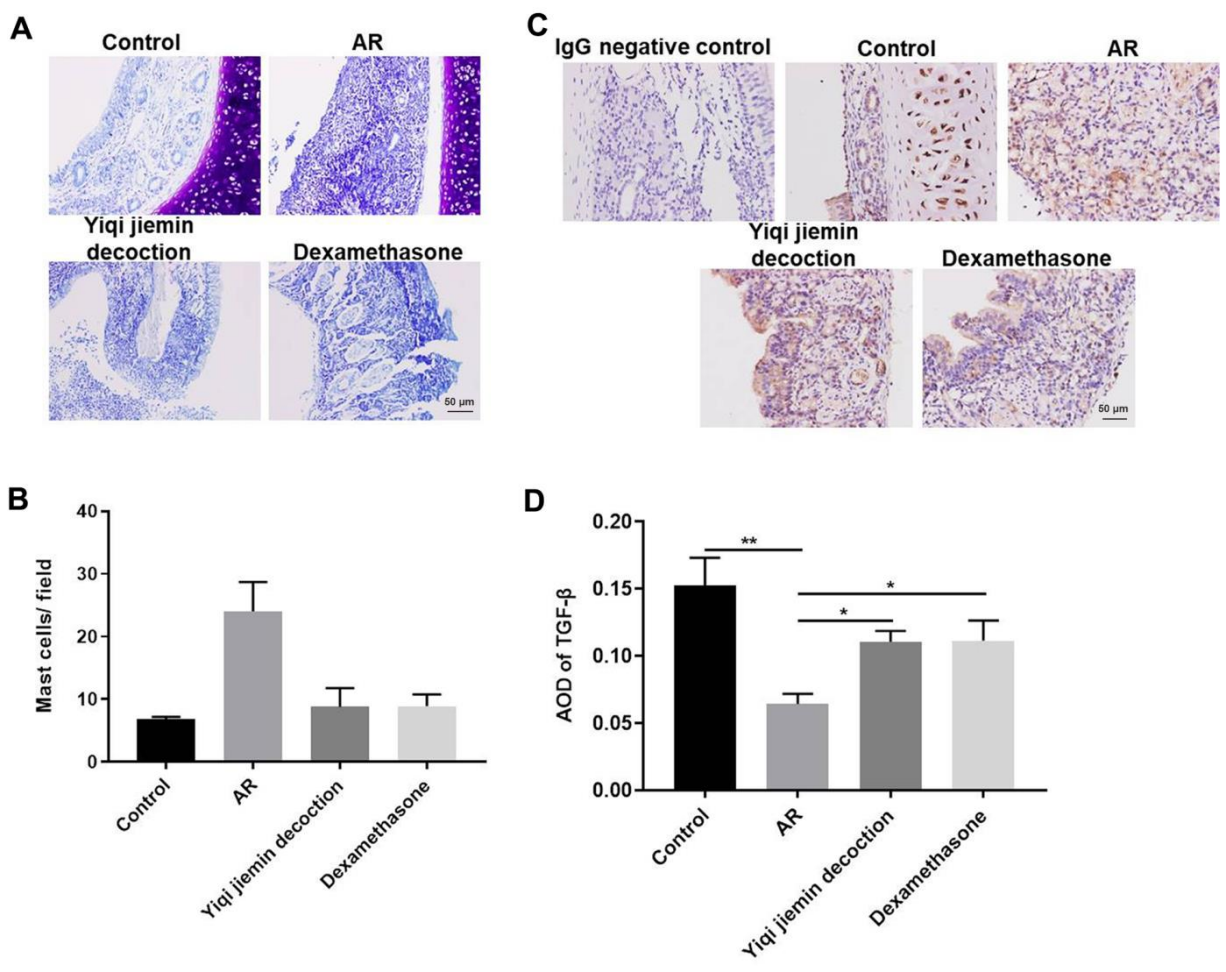
**YJD treatment reduces inflammation in the nasal mucosal tissues of AR model guinea pigs.** (**A**) Toluidine blue staining of nasal mucosal samples from control, AR model, AR model plus YJD, and AR model plus dexamethasone groups. (**B**) The histogram plots show the percentage of mast cells in the nasal mucosal samples from the control, AR model, AR model plus YJD, and AR model plus dexamethasone groups based on toluidine blue staining. The samples were analyzed independently by 3 pathologists based on five different staining views. *P < 0.05; **P < 0.01; one-way ANOVA. (**C**) IHC staining of nasal mucosal samples from control, AR model, AR model plus YJD treatment, and AR model plus dexamethasone treatment groups using the anti-TGF-β antibody (magnification: 200×). IgG staining was used as negative control. (**D**) The histogram plots show average optical density (AOD) of TGF-β expression in the nasal mucosal samples from control, AR model, AR model plus YJD, and AR model plus dexamethasone groups. *P < 0.05; **P < 0.01; one-way ANOVA.

Mast cells (MC) play an essential role in AR pathogenesis [23]. Therefore, we analyzed the status of MC infiltration in the nasal mucosal tissues by toluidine blue staining. Mast cell infiltration was significantly higher in the nasal mucosa of the AR model group compared to the control group, but was significantly reduced in the YJD and dexamethasone treatment groups (Figure 2A, 2B). We then determined the levels of the anti-inflammatory cytokine, TGF-β, in the nasal mucosa. The levels of TGF-β were significantly lower in the AR model group compared to the controls, but were moderately increased in the YJD and dexamethasone treatment groups (Figure 2C). IHC analysis showed that TGF-β staining in the nasal mucosal samples from the YJD and dexamethasone treatment groups was significantly higher than the model group (Figure 2D). Taken together, these data demonstrated that YJD treatment decreased mast cell infiltration and increased the levels of TGF-β in the nasal mucosa of AR model guinea pigs.

### YJD decreases inflammation and restores the balance of Th1/Th2 immune cell responses in AR model guinea pigs

**Figure 3:**
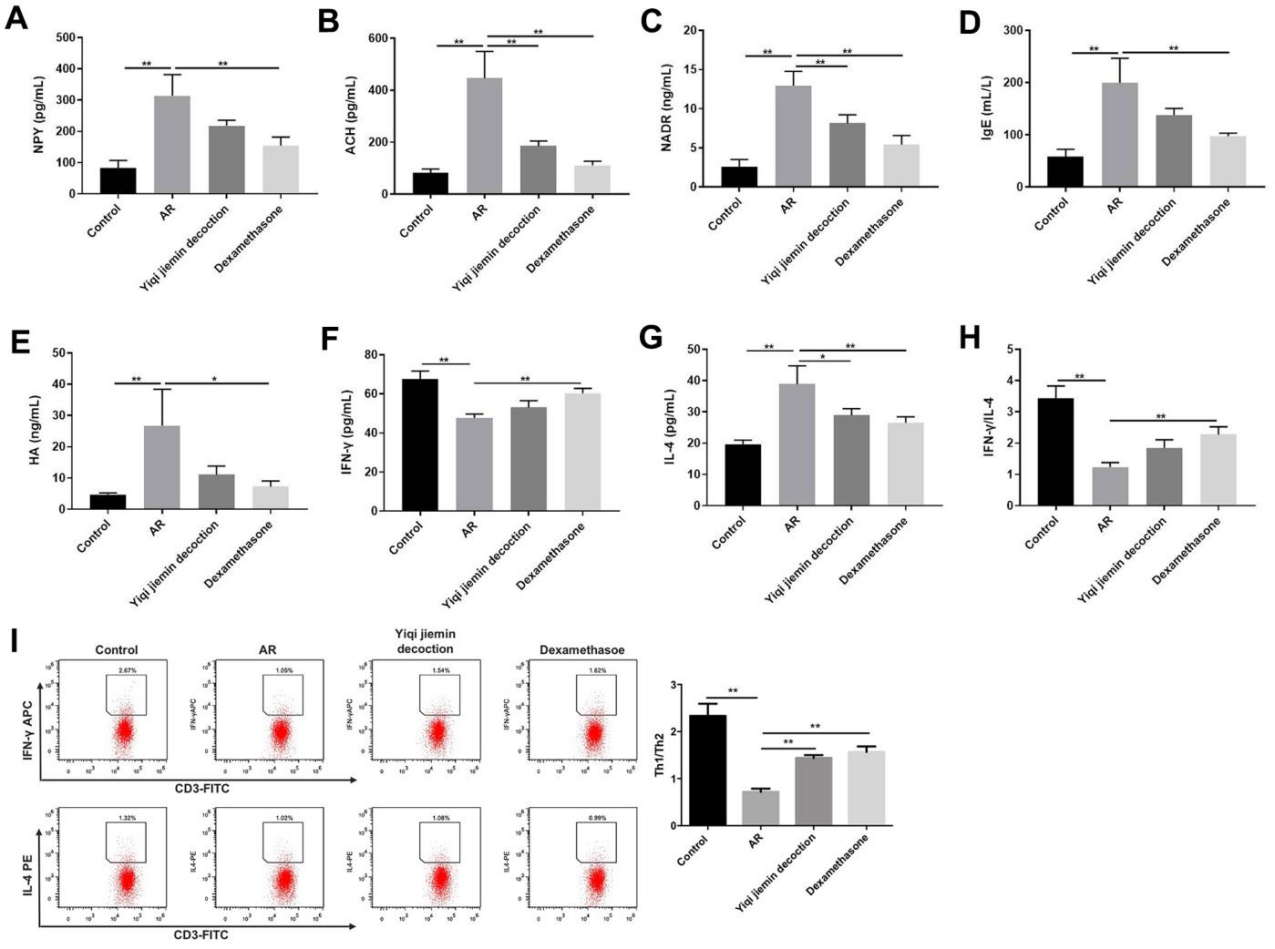
**YJD reduces serum levels of pro-inflammatory factors in the AR model guinea pigs and restores the balance of Th1/Th2 immune responses.** ELISA assay results show the serum levels of NP-γ (**A**), ACH (**B**), NADR (**C**), IgE (**D**), HA (**E**), IFN-γ (**F**) and IL-4 (**G**) in the control, AR model, AR model plus YJD, and AR model plus dexamethasone groups. (**H**) The histogram plots show the IFN-γ/IL-4 ratio based on the serum levels of IFN-γ and IL-4 in the control, AR model, AR model plus YJD, and AR model plus dexamethasone groups. (**I**) Flow cytometry was used to detect the CD3^+^IFN-γ ^+^ (Th1) cell percentage and CD3^+^IL-4^+^ (Th2) cell percentage respectively. *P < 0.05; **P < 0.01; one-way ANOVA.

Next, we measured the serum levels of pro-inflammatory mediators such as HA, NP-γ, ACH, NADR, and IgE by ELISA. The serum levels of HA, NP-γ, ACH, NADR, and IgE were significantly upregulated in the AR model group compared to the control group, but were significantly reduced in the YJD and dexamethasone treatment groups (Figure 3A–3E). Furthermore, we analyzed the status of the T helper 1 (Th1) and T helper 2 (Th2) cell responses by measuring the levels of IFN-γ or IL-4, respectively. The levels of IFN-γ were significantly lower and the levels of IL-4 were significantly higher in the AR model group compared to the control group, but these effects were reversed by YJD and dexamethasone treatments (Figure 3F, 3G). The IFN-γ/IL-4 ratio was lower in the AR group, but this ratio was reversed by YJD and dexamethasone treatments (Figure 3H). In addition, the results of flow cytometry assay showed that the Th1/Th2 ratio was lower in the AR group compared to the control group, but that effect was reversed by YJD and dexamethasone treatments (Figure 3I). These results demonstrated that YJD decreased inflammation and normalized Th1/Th2 cell responses in the AR model guinea pigs.

### YJD improves cellular metabolism in the AR model guinea pigs

**Figure 4:**
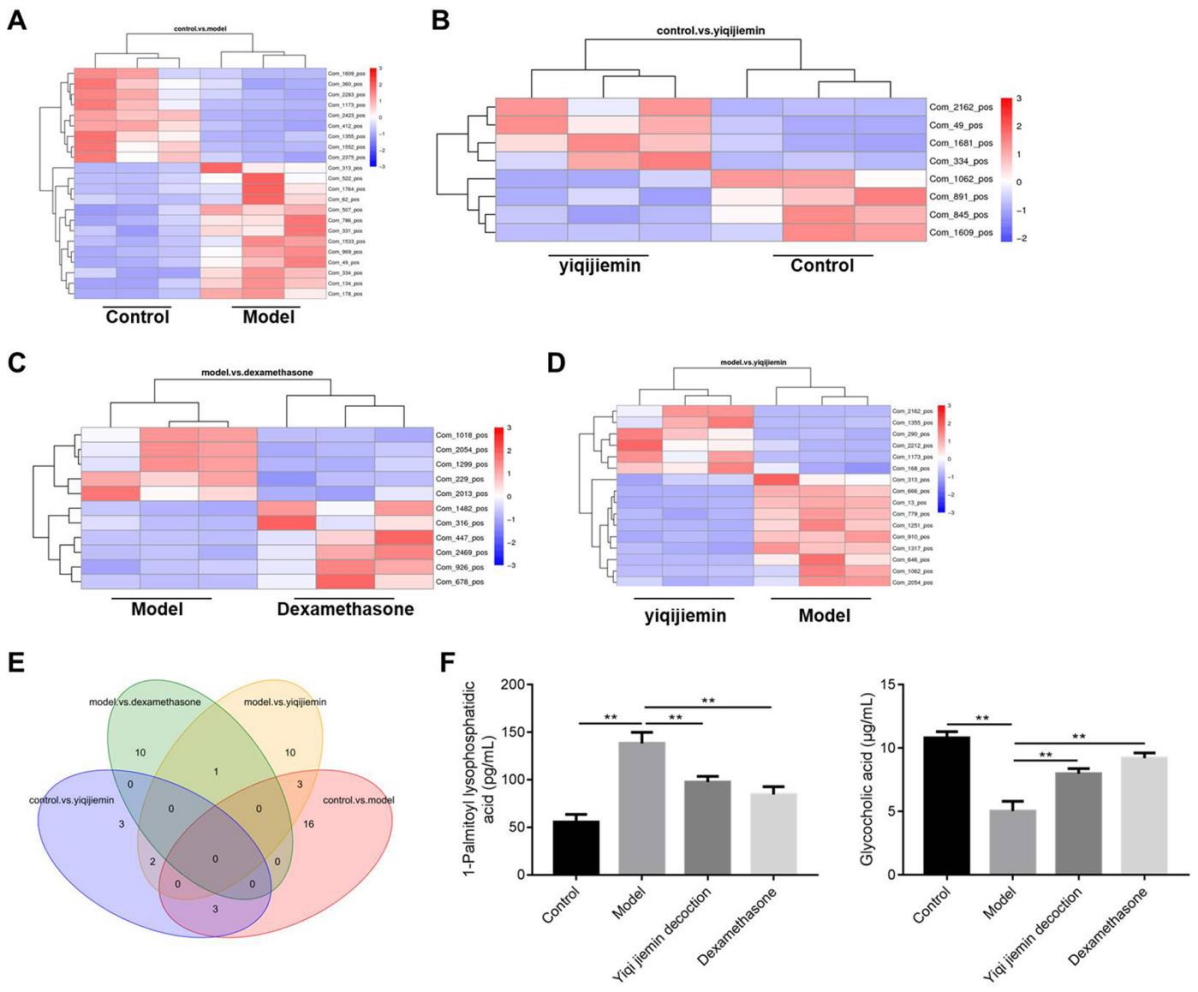
**YJD treatment reverses serum metabolite changes in the AR model guinea pigs.** (**A**–**D**) Heat map shows clustering of various metabolites as analyzed by LC-HRMS in the control vs. AR model, AR model plus YJD vs. control, AR model vs. AR model plus dexamethasone, and AR model vs. AR model plus YJD groups. (**E**) Venn diagram shows the common metabolites that are differentially expressed when comparing control vs. AR model, AR model plus YJD vs. control, AR model vs. AR model plus dexamethasone, and AR model vs. AR model plus YJD groups. (**F**) ELISA assay results show the levels of LPA and GCA metabolites in the serum of control, AR model, AR model plus YJD, and AR model plus dexamethasone groups.

We then used liquid chromatography coupled to high resolution mass spectrometry (LC-HRMS) to investigate YJD-induced metabolic changes and identified alterations in the levels of 57 metabolites. The levels of 9 metabolites were significantly higher and the levels of 13 metabolites were significantly reduced in the AR model group compared to the control group (Figure 4A–4D). In comparison with the model group, the levels of 10 metabolites were significantly higher and those of 6 metabolites were significantly lower in the YJD group (Figure 4A–4D). The heat maps (Figure 4A–4D) and dot plots (Supplementary Figure 1A–1D) show the levels of various metabolites in the four groups of guinea pigs. Venn diagram showed that the levels of 3 metabolites, namely, Com_1173_pos (4,4-Difluoropregn-5-ene-3,20-dione), Com_1355_pos (Glycocholic acid; GCA) and Com_313_pos (1-Palmitoyl lysophosphatidic acid; LPA) varied consistently among the 4 groups of guinea pigs (Figure 4E). We did not observe statistically significant differences in the expression of these 3 metabolites between the model and dexamethasone groups (Figure 4E). However, LPA levels were significantly higher and GCA levels were significantly lower in the AR group compared to the control group, but these effects were reversed by YJD and dexamethasone treatments (Figure 4F).

**Figure 5:**
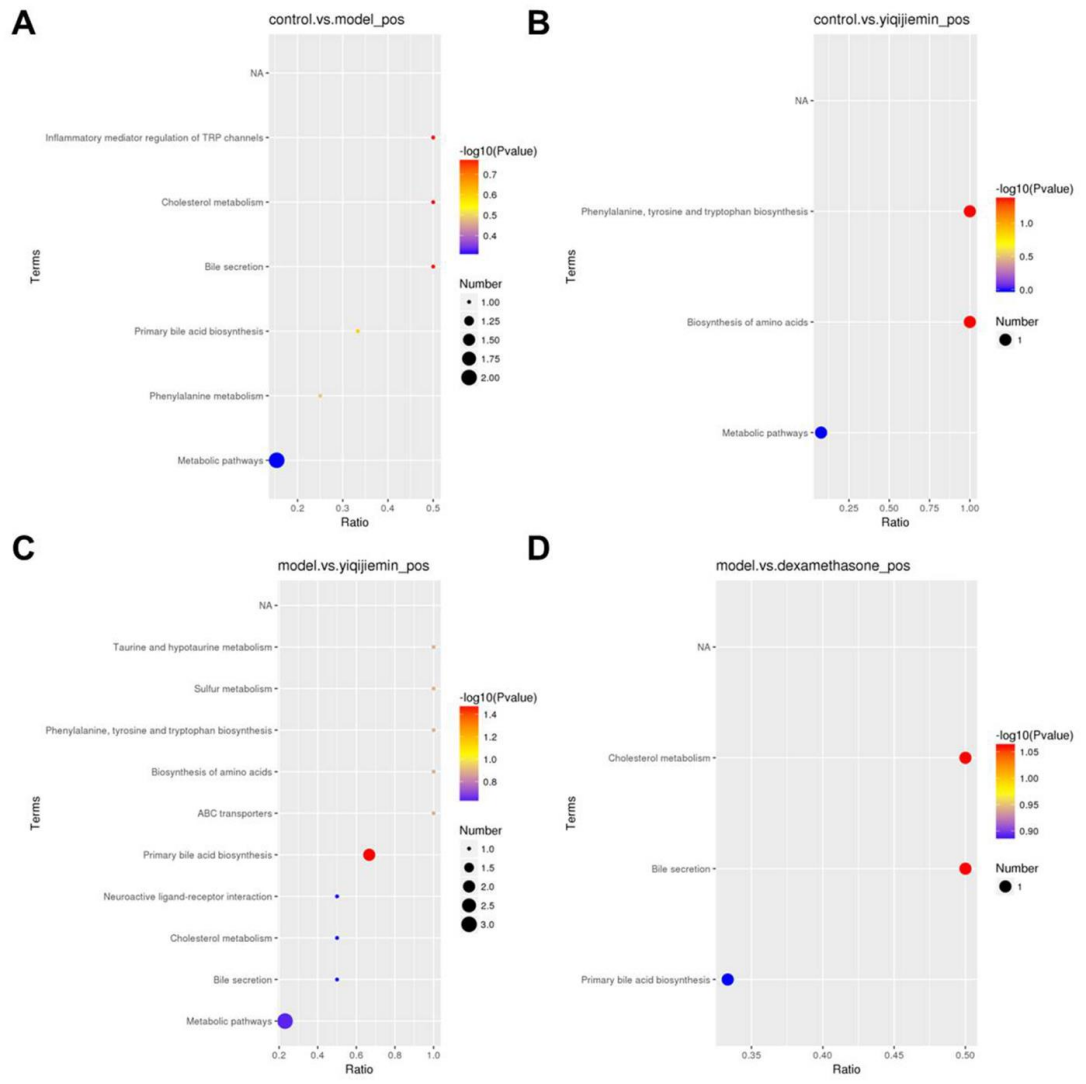
**KEGG pathway enrichment analysis of differentially expressed serum metabolites in the AR model mice treated with YJD and dexamethasone.** (**A**–**D**) KEGG enrichment analysis results show the top enriched KEGG pathways based on the differentially expressed serum metabolites between (**A**) control and AR model, (**B**) control and AR model plus YJD, (**C**) AR model and AR model plus YJD, and (**D**) AR model and AR model plus dexamethasone groups.

KEGG pathway enrichment analysis of the differentially expressed metabolites showed that metabolic pathway was enriched in the AR and YJD groups compared to the control group (Figure 5A, 5B). Moreover, metabolic pathway was also enriched in the YJD group compared to the AR group (Figure 5C). However, primary bile acid biosynthesis pathway was enriched in the dexamethasone treatment group compared to the AR group (Figure 5D). These results demonstrated that YJD alleviated AR by improving cellular metabolism.

### RNA-seq analysis shows that YJD inhibits expression of pro-inflammatory cytokines in the AR model guinea pigs

We then performed RNA-seq analyses to determine genome-wide transcriptome changes in the nasal mucosal tissues among the four groups of guinea pigs.

**Figure 6:**
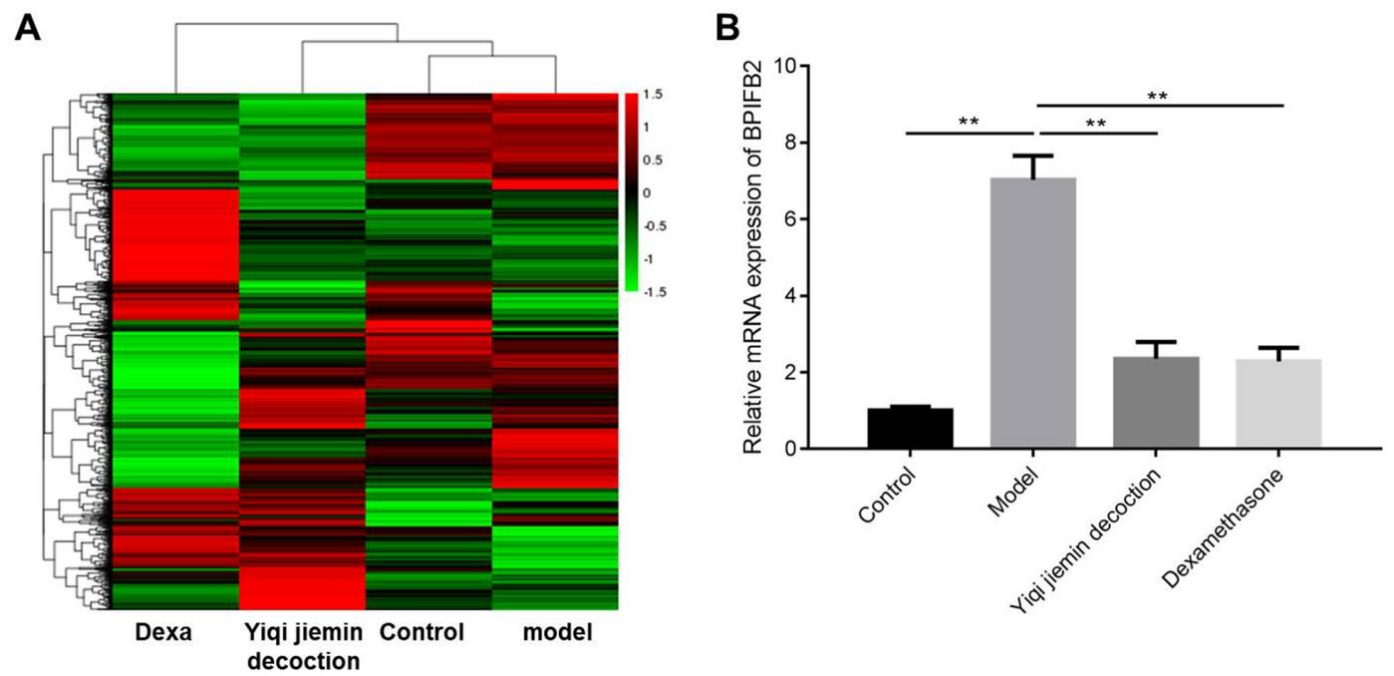
**Identification of differentially expressed genes (DEGs) in the nasal mucosal tissues of AR model guinea pigs treated with YJD or dexamethasone.** (**A**) Heat map shows differentially expressed genes (DEGs) in the nasal mucosal samples from the control, AR model, AR model plus YJD, and AR model plus dexamethasone groups. The color code (green to red) denotes the relative expression levels of the DEGs (high or low). (**B**) QRT-PCR analysis shows BPIFB2 mRNA levels in the nasal mucosal samples from the control, AR model, AR model plus YJD, and AR model plus dexamethasone groups. *P < 0.05; **P < 0.01; one-way ANOVA.

We used two-fold cut-off criterion to identify differentially expressed genes (DEGs) and identified 98 upregulated and 150 downregulated genes in the AR group compared to the control group (Figure 6A and Supplementary Figure 2A). We also identified 272 upregulated and 386 downregulated genes in the YJD group compared to the AR group; 352 genes were upregulated and 396 were downregulated in the YJD group compared to the control group (Figure 6A and Supplementary Figure 2B–2D). Moreover, 2123 genes were upregulated and 1691 genes were downregulated in the AR group compared to the dexamethasone group (Figure 6A and Supplementary Figure 2C). The expression of bacterial permeability-increasing (BPI)-fold containing family B, member 2 (BPIFB2) was significantly upregulated in the AR group compared to the control group; but was significantly reduced in the YJD and dexamethasone groups (Figure 6A). QRT-PCR analysis confirmed that BPIFB2 mRNA expression was significantly upregulated in the AR group compared to the control group; but was significantly reduced in the YJD and dexamethasone groups (Figure 6B).

**Figure 7:**
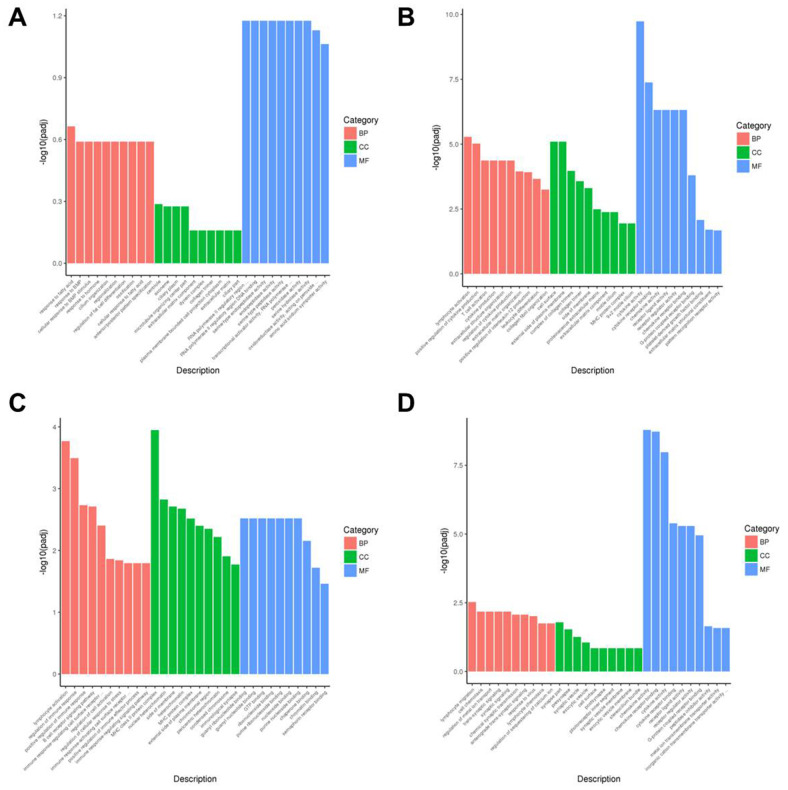
**Gene ontology enrichment analyses of DEGs in the nasal mucosal tissues of AR model guinea pigs treated with YJD or dexamethasone.** The top 30 enriched GO terms related to the biological process (BP), cellular component (CC), and molecular function (MF) categories based on the analyses of DEGs for the (**A**) control vs. AR model, (**B**) control vs. AR model plus YJD, (**C**) AR model vs. AR model plus dexamethasone, and (**D**) AR model vs. AR model plus YJD groups.

Next, we performed gene ontology (GO) analysis and showed that DEGs in the AR model group were highly enriched in transcription regulation (MF), centriole functions (CC), and fata cell regulation (BP) (Figure 7A and Supplementary Figure 3A). DEGs in the YJD group were enriched in cytokine activity (MF), cell surface (CC), and cytokine production (BP) (Figure 7B, 7D and Supplementary Figure 3B, 3D). Moreover, DEGs in the dexamethasone group were mainly enriched in immunological synapse (MF) as well as guanyl ribonucleotide binding and GTP-binding (BP) (Figure 7C and Supplementary Figure 3C).

**Figure 8:**
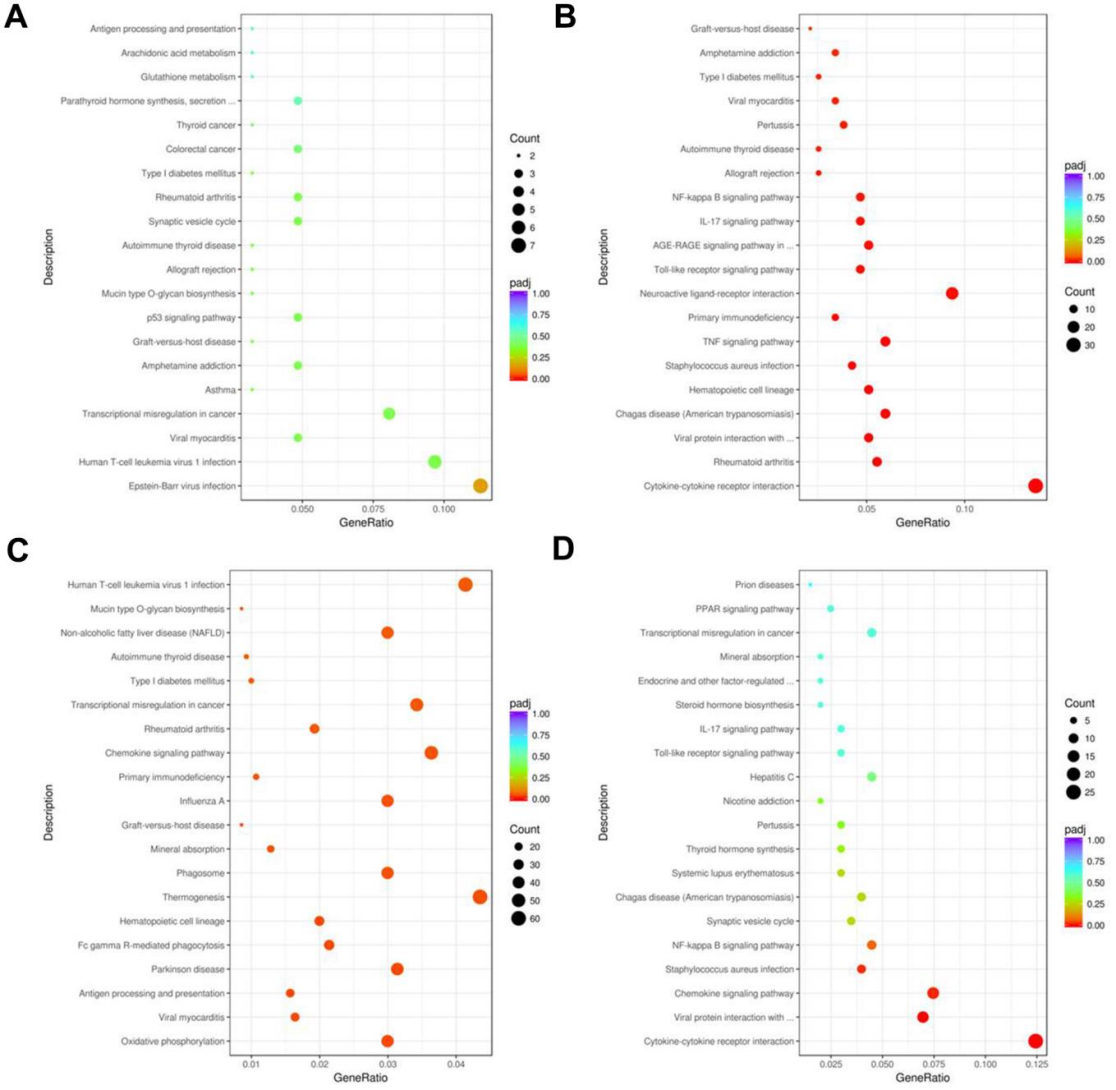
**KEGG pathway enrichment analyses of DEGs in the nasal mucosal tissues of AR model guinea pigs treated with YJD or dexamethasone.** Top 20 KEGG pathways based on the functional enrichment analysis of DEGs for the (**A**) control vs. AR model, (**B**) control vs. AR model plus YJD, (**C**) AR model vs. AR model plus dexamethasone, and (**D**) AR model vs. AR model plus YJD groups.

KEGG pathway enrichment analysis demonstrated that the top 20 significant pathways in the AR, YJD, and dexamethasone groups were involved in cytokine interactions, cytokine binding, and cytokine production (Figure 8A–8D). DEGs in the YJD group were enriched in pathways related to cytokine-cytokine receptor interactions, NF-kB, and IL-17 signaling (Figure 8B, 8D). DEGs in the dexamethasone group were enriched in pathways regulating thermogenesis and chemokine signaling (Figure 8C). These data showed that YJD alleviated AR by regulating cytokine secretion and were consistent with our previous data regarding measurement of serum cytokine levels (Figure 3G, 3H). Significantly, the expression of p-NF-kB p65 were upregulated in the AR model group; however, that effect was reversed by YJD and dexamethasone treatments (Supplementary Figure 4A). In addition, the levels of IL-6, TNF-α and IL-1β were upregulated in the AR model group; however, these levels were reversed by YJD and dexamethasone treatments (Supplementary Figure 4B). These data suggested that YJD could suppress inflammation in a guinea pig model via inactivating NF-kappaB pathway.

## DISCUSSION

Traditional Chinese Medicines (TCMs) have been used for many centuries to treat multi-factorial diseases including AR. YJD is a widely used TCM regime to treat inflammatory diseases in China [16, 18, 19, 24]. In this study, we confirmed that YJD alleviated OVA-induced AR in the guinea pig model by suppressing inflammation and improving cellular metabolism. Therefore, our study suggested that YJD was a promising treatment for AR patients.

The nasal symptoms of AR are mediated by histamine and other inflammatory mediators, which are secreted by activated MC, eosinophils and basophils [25]. Epithelial barrier dysfunction, allergic sensitization, and hyperactivation of mast cells and eosinophils are involved in the pathogenesis of AR [26]. MC-derived mediators collectively induce acute-phase clinical symptoms of AR by enhancing vascular leakage, bronchospasms, and parasympathetic reflexes linked to the activation of nociceptive neurons [27, 28]. IL-4 mediated IgE production by the B cells is a critical step in the allergic cascade [29]. Th2 cells play an important role in the development of IgE-related diseases such as AR [30]. Th1 cytokines such as IFN-γ suppress the Th2 cell responses and are used in the treatment of IgE-related allergic diseases such as AR [31, 32]. Furthermore, the ratio of IFN-γ/IL-4 levels in the serum is used to determine the balance between Th1 and Th2 immune responses [32]. In this study, our data demonstrated that YJD ameliorated AR symptoms by reducing the secretion of inflammatory cytokines and rebalancing the Th1/Th2 cytokine ratio. These results further demonstrated that YJD suppressed inflammation.

Transcriptomic approaches have been extensively used to identify mechanisms of several drugs [33]. Liquid chromatography coupled with mass spectrometry (LC-MS) and principal component analysis has been used to successfully characterize structures of various biological molecules and their interactions with several drugs [33]. In this study, we showed that both YJD and dexamethasone alleviated AR progression by inhibiting the secretion of inflammatory cytokines. The analysis of metabolite data demonstrated that the serum levels of LPA and GCA were significantly associated with AR. LPA promotes eosinophil recruitment in response to allergen challenge. Moreover, LPA stimulates IL-6 and IL-8 expression via the LPA receptor [34]. GCA plays a protective role in gut mucosal damage by suppressing inflammatory signaling pathways [35]. Our data demonstrated that LPA and GCA were enriched in the metabolic pathways related to inflammation. We further showed that YJD suppressed progression of inflammatory AR disease by partially reversing the levels of metabolites such as LPA and GCA. Moreover, RNA-Seq data analysis revealed differential transcription patterns upon YJD treatment. We identified BPIFB2 as an important diagnostic biomarker for AR and YJD treatment efficacy. BPIFB2 is a protein with strong homology to the BPI protein family in mammals [36]. Previous studies have shown that BPIFB2 is a distinct marker of inflammation; high levels of BPI proteins are released by different pathogens during acute pneumonia; BPIFB2 is also associated with ORF3 regulation in Hepatitis E virus- (HEV-) mediated hepatitis [36]. Therefore, we postulated that YJD suppressed inflammation in AR via BPIFB2. Our findings suggested that LPA, GCA, and BPIFB2 were promising diagnostic biomarkers and therapeutic targets for AR. Functional enrichment analysis of the DEGs showed that YJD treatment significantly altered cytokine secretion in AR. However, further investigations are necessary to confirm our results in human AR patients.

In this study, we found that YJD could suppress inflammation in a guinea pig model via inactivating NF-kappaB pathway. However, the mechanism by which YJD alleviated allergic rhinitis remains largely unclear. It has been shown that inhibition of Wnt/β-catenin and JAK1/STAT3 signalings could suppress the development of allergic rhinitis [37, 38]. Thus, further study is needed to investigate whether YJD could alleviate allergic rhinitis via regulating Wnt/β-catenin or JAK2/STAT3 signaling pathways.

In conclusion, integrated metabolomic and transcriptomic analysis demonstrated that YJD alleviated AR in the *in vivo* guinea pig model by suppressing inflammation and improving cellular metabolism. We also demonstrated that BPIFB2 was a potential diagnostic biomarker and therapeutic target in AR patients.

## MATERIALS AND METHODS

### Animals

We purchased 8-week-old guinea pigs (body weight: 400 ±20 g) from Wuhan Wanqianjiaxing Biotechnology Company (Wuhan, China). They were housed under standard conditions (Temperature: 23 ± 2° C, 12 h light-dark cycle; and 65% humidity) with free access to standard food and water. All animal protocols were approved by the Ethics Committee of Dongzhimen Hospital, The First Affiliated Hospital of Beijing University of Chinese Medicine (Beijing, China), and were carried out according to the National Institute of Health (NIH) guidelines for the care and use of animals.

### Preparation and administration of YJD

YJD was prepared by soaking a powdered mixture of *Astragalus* (30 g), bran-fried *Atractylodes rhizoma* (10 g), *Radix saposhnikoviae* (10 g), *Ephedrae* (10 g), cinnamon sticks (10 g), peony root (12 g), dried ginger (10 g), *Schisandra* (10 g), *Asarum* (3 g), *Magnoliae flos* (10 g), *Radix bupleuri* (10 g), *Periostracum cicada* (10 g), and *Radix glycyrrhizae* preparata (10 g) in water (1:12 weight/volume ratio) for 50 min. Subsequently, the mixture was boiled for 60 min and filtered. The dregs were then added to water, and the above steps were repeated [39].

### Establishment of the AR model mice

The AR model guinea pigs were established as described previously with minor modifications [18, 19]. In brief, guinea pigs were sensitized for 15 consecutive days with intraperitoneal injections of 750 μL OVA solution, which consisted of 2 mg ovalbumin (Sigma-Aldrich, USA; cat. no. S7951) emulsified in a 1mg/ml Al (OH)_3_ solution prepared in 10 mL physiological saline. Then, the sensitized guinea pigs were administered daily with 1% w/v OVA in physiological saline into their nasal cavities from days 15 to 21. After the last OVA treatment, the AR symptoms were evaluated for 30 min using the scoring criteria, which included estimating the frequency of sneezing, nose-rubbing, and the extent of rhinorrhea. The guinea pigs with more than 5 points according to the scoring criteria were considered as AR models.

Guinea pigs administered with physiological saline for 2 weeks were used as the control group (n=8). The AR model guinea pigs were then randomly divided into three groups (n=8/group): (1) AR model group (administered orally for 2 weeks with 10 mL physiological saline/kg body weight), (2) AR model plus YJD group (administered orally for 2 weeks with 0.155 YJD package), and (3) AR model plus dexamethasone group (administered orally for 2 weeks with 1.53mg/kg dexamethasone). Dexamethasone was purchased from Shanghai Yuanye Bio-Technology Company. After 2 weeks, the guinea pigs were sacrificed. The nasal tissues were harvested and tested for various parameters.

### Histology

The guinea pigs were euthanized using isoflurane and decapitated for blood collection. The nasal tissues were harvested, fixed in 4% paraformaldehyde (Sigma-Aldrich, USA; cat. no. 158127) for 2 days, gradually dehydrated with graded series of alcohol, and embedded in paraffin. Then, 4-6 μm serial sections of the paraffin-embedded nasal tissue samples were cut and mounted onto 3-ammonia propyl-3-ethoxy silane (APES)-treated slides. After deparaffinization, the sections were stained with hematoxylin and eosin (H&E; Sigma-Aldrich, USA) for general histology, toluidine blue stain (Sinopharm group, China) for estimating mast cells, and Wright’s-Giemsa stain (Abcam Cambridge, MA, USA; cat. no. ab245888) for estimating eosinophils. The stained sections were examined and photographed under a light microscope.

### Immunohistochemistry

The nasal mucosal tissue sections were deparaffinized and rehydrated with graded series of ethanol. Then, antigen retrieval was performed. The sections were then blocked for 1h with 3% bovine serum albumin (BSA, Thermo Fisher Scientific; cat. no. 37520) followed by overnight incubation with anti-TGF-β primary antibody (cat. no. PAA124Gu01; USCN Life Science Incorporated, China) at 4° C in a humidified chamber. The sections were then incubated with a secondary antibody. The sections were then developed with diaminobenzidine, counterstained by hematoxylin, washed 3 times with PBS, and photographed under a light microscope. TGF-β expression was quantified using the Image Pro Plus 6.0 analysis system (Media Cybernetics, USA). Briefly, 10 digital images were captured at 1360×1024 pixel resolution and 400× magnification. Then, the size of TGF-β-positive area and the integrated optical density (IOD) in each area was evaluated. Finally, average optical density (AOD = IOD/Area) was calculated to determine TGF-β expression values for each sample.

### ELISA assay

We centrifuged blood serum samples at 300 rpm for 10 min at 4° C and used the supernatants to measure IL-6, TNF-α, IL-1β, NP-γ, HA, ACHI, IL4, IFNγ, NADR, and IgE levels. ELISA assay kits for estimating NP-γ (cat. no. CEA879Gu), HA (cat. no. CEA927Ge), ACH (cat. no. CEA912Ge), IL4 (cat. no. SEA077Gu), IFNγ (cat. no. SEA049Gu), IL-6 (cat. no. SEA079Gu), TNF-α (cat. no. SEA133Gu) and IL-1β (cat. no. SEA563Gu) were purchased from USCN Life Science Inc. (Wuhan, China; http://www.uscnk.com), whereas, those used for estimating NADR (cat. no. E03N0013) and IgE (cat. no. E05I0037) were purchased from Bluegene (China). Glycocholic acid and 1-Palmitoyl lysophosphatidic acid levels in the blood serum samples were analyzed using the corresponding ELISA kits purchased from Buya-TFK (USA). All experiments were performed according to the manufacturer's instructions.

### Real-time polymerase chain reaction (qRT-PCR)

Total RNA was extracted from nasal tissues using TRIzol reagent (cat. no. 15596018; Thermo Fisher Scientific, USA). CDNA synthesis was performed using total RNA (1μg) samples with the Verso cDNA Kit (Thermo Fisher Scientific, USA). Then, q-PCR was performed to quantify the relative expression levels of BPIFB2. The temperature conditions were as follows: 95° C for 3 min, followed by 40 cycles at 95° C for 10 sec, 58° C for 30 sec and 72° C for 30 sec. GAPDH was used as the internal control. The q-PCR primers were: BPIFB2-F: 5’-CAGCACCTGTTTGACTGTGC-3’; BPIFB2-R: 5’-GCTGCACCTTGAGTACCAGT-3’; GAPDH-F: 5’-TTCTACCCACGGCAAGTTCC-3’; GAPDH-R: 5’-CCAGCATCACCCCACTTGAT-3’. The relative expression of BPIFB2 was calculated using the 2^-ΔΔCt^ method.

### Flow cytometry assay

Flow cytometry assay was carried out as described by Ren et al. [40]. After incubation, cells were analyzed in a FACScalibur flow cytometer (BD Biosciences).

### RNA sequencing

We performed paired-end RNA sequencing (RNA-seq) using 2 μg of total RNA from the nasal mucosal tissues. The quality and quantity of the RNA samples was determined using the Agilent 2100 bioanalyzer. The RNA sequencing library was prepared using the UltraTM RNA Library Prep Kit (NEB, USA) according to the manufacturer’s instructions and sequenced with the Illumina Hiseq 4000 machine. The sequencing reads were processed and aligned to the human genome assembly (hg19) using TopHat (version 2.0) and Bowtie2.

### Metabolomics

We obtained 2 mL of femoral artery blood from all guinea pigs after the last treatment and incubated them at room temperature for 30 min. The samples were then centrifuged at 3000 rpm for 10 min and obtained the supernatant. The supernatant was incubated with 500 μL methanol solution (80% methanol, 0.1% formic acid) at 4° C for 5 min, centrifuged at 3000 rpm for 10 min at 4° C, diluted with ultrapure water to obtain a final methanol concentration of 60%, and filtered through a 0.22 μm filter. The samples were analyzed by LC-HRMA/MS using a Dionex UltiMate 3000 chromatographic system (CTC Analytics AG, Switzerland) coupled to a TF Q-Exactive mass spectrometer equipped with a heated electrospray ionization (HESI)-II source.

### Gene ontology and KEGG pathway enrichment analysis

Functional enrichment analysis was performed to determine gene ontology (GO) terms and KEGG pathways associated with the differentially expressed genes (DEGs). Gene ontology (GO) analysis was performed using the GOseq R package. Kyoto Encyclopedia of Genes and Genomes (KEGG) pathway analysis was performed using the clusterprofile software.

### Western blot assay

The protein lysates were separated by SDS-PAGE and then transferred onto polyvinylidene difluoride (PVDF) membranes. Later on, the membranes were incubated with primary antibodies at 4° C overnight, and then incubated with the corresponding secondary antibodies for 1 h. After that, the membrane was visualized with enhanced chemiluminescence reagent (Thermo Fisher Scientific). Rabbit polyclonal p-NF-κB p65 (cat. no. #3033, Cell Signaling Technology), NF-κB p65 (cat. no. #8242; Cell Signaling Technology) and β-actin (cat. no. #12262; Cell Signaling Technology) were used at a dilution of 1:1,000.

### Statistical analysis

All statistical data was expressed as means ± SD of three independent experiments. SPSS 13.0 software was used for statistical analyses. The differences between groups were estimated using one-way analysis of variance (ANOVA) followed by Dunnett’s test and LSD analysis of variance. All comparisons were made relative to the corresponding controls. P < 0.05 was considered statistically significant.

## Supplementary Material

Supplementary Figures
