# Supplementary material for: Yiqi Jiemin decoction alleviates allergic rhinitis in a guinea pig model by suppressing inflammation, restoring Th1/Th2 balance, and improving cellular metabolism
Source: Aging (Albany NY). 2021 Jul 27;13(14):18423–41. doi: 10.18632/aging.203292 (PMC8351690; doi:10.18632/aging.203292)
Supplement: Supplementary Figures [file aging-13-203292-s001.pdf]

SUPPLEMENTARY FIGURES

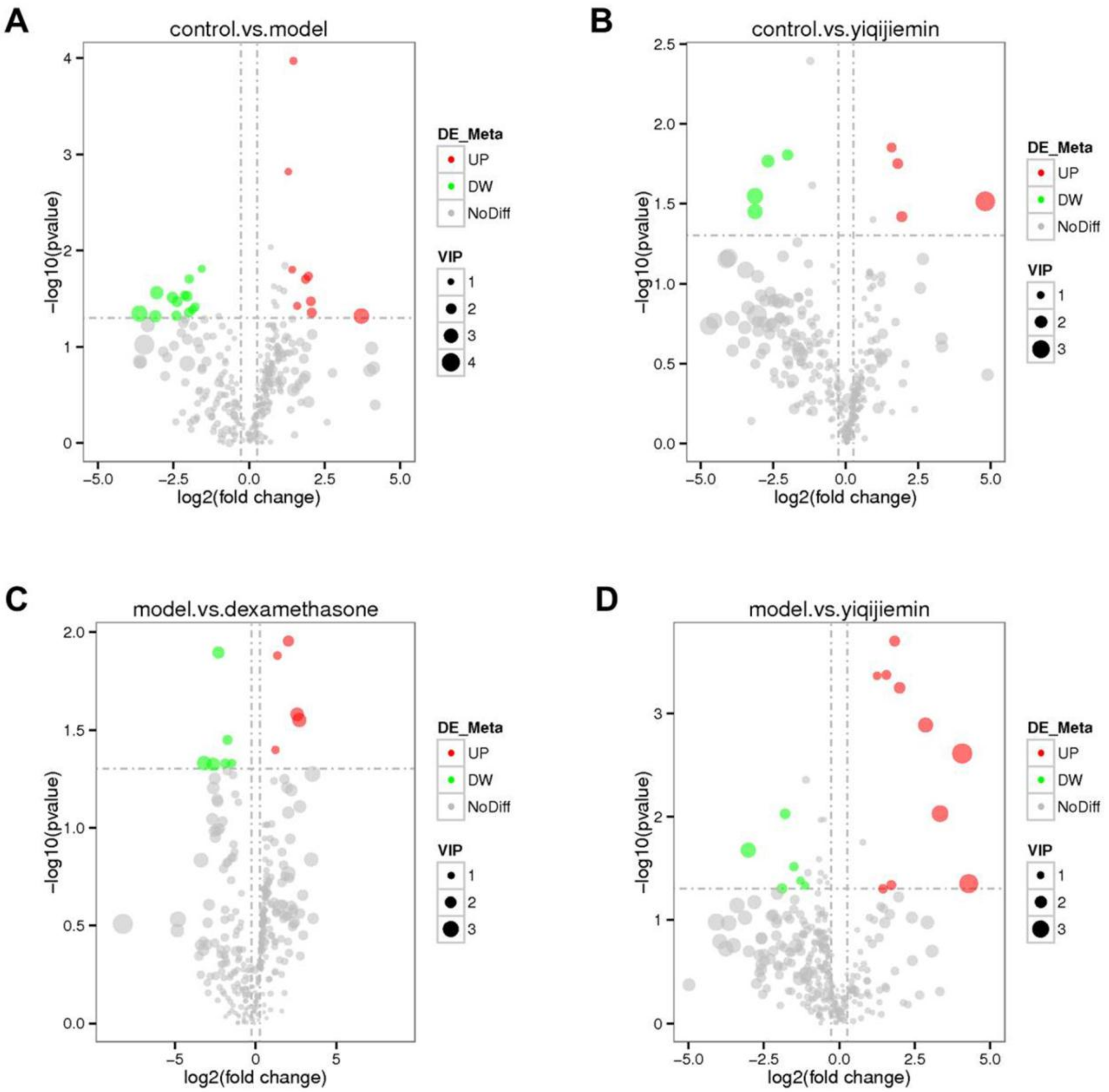

Supplementary Figure 1. Identification of differentially expressed serum metabolites. Volcano plots show differentially expressed metabolites between (**A**) control vs. AR model, (**B**) control vs. AR model plus YJD, (**C**) AR model vs. AR model plus dexamethasone, and (**D**) AR model vs. AR model plus YJD groups.

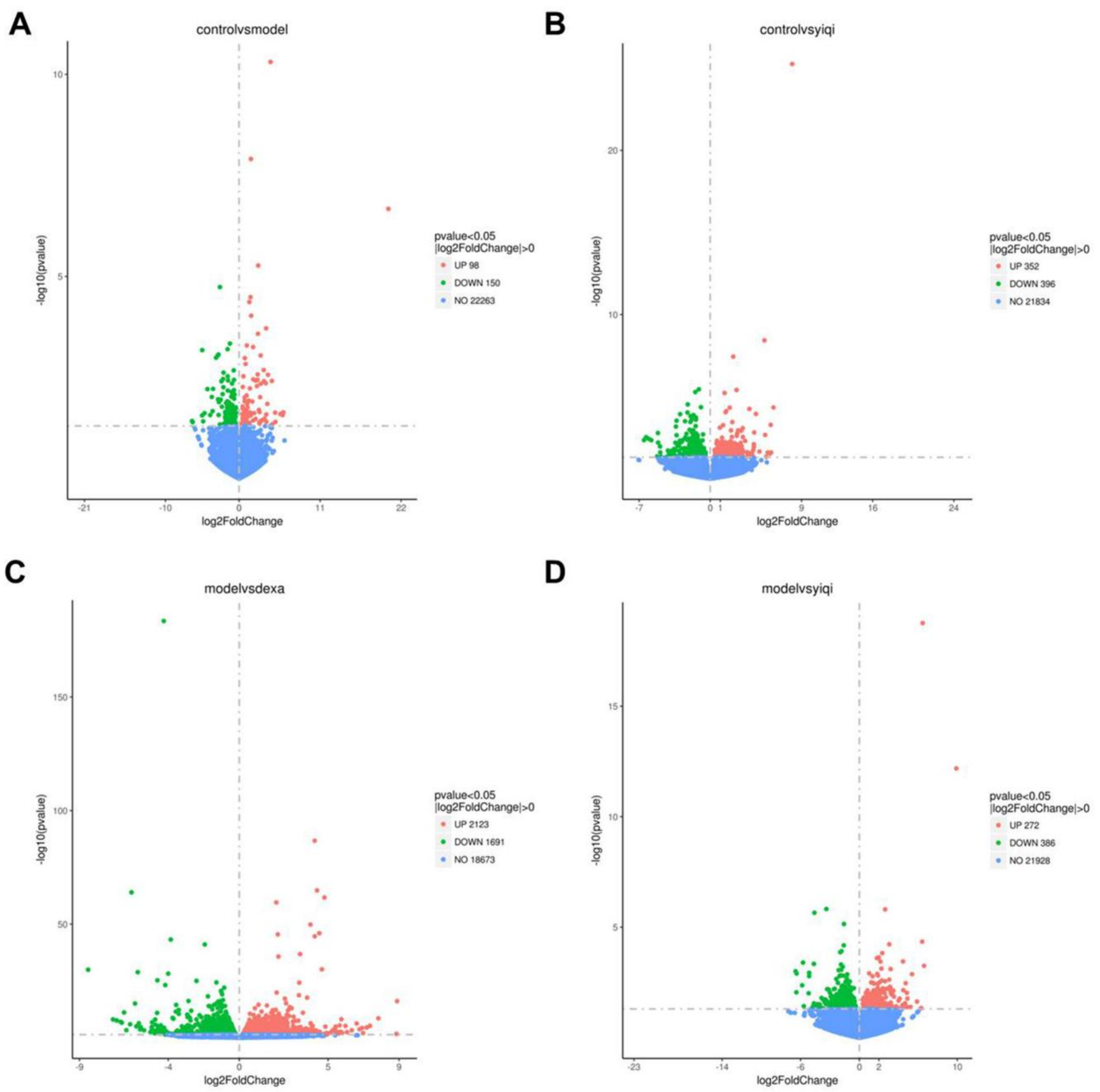

Supplementary Figure 2. Identification of differentially expressed genes in the nasal mucosal tissues. Volcano plots show differentially expressed genes (DEGs) between (**A**) control vs. AR model, (**B**) control vs. AR model plus YJD, (**C**) AR model vs. AR model plus dexamethasone, and (**D**) AR model vs. AR model plus YJD groups.

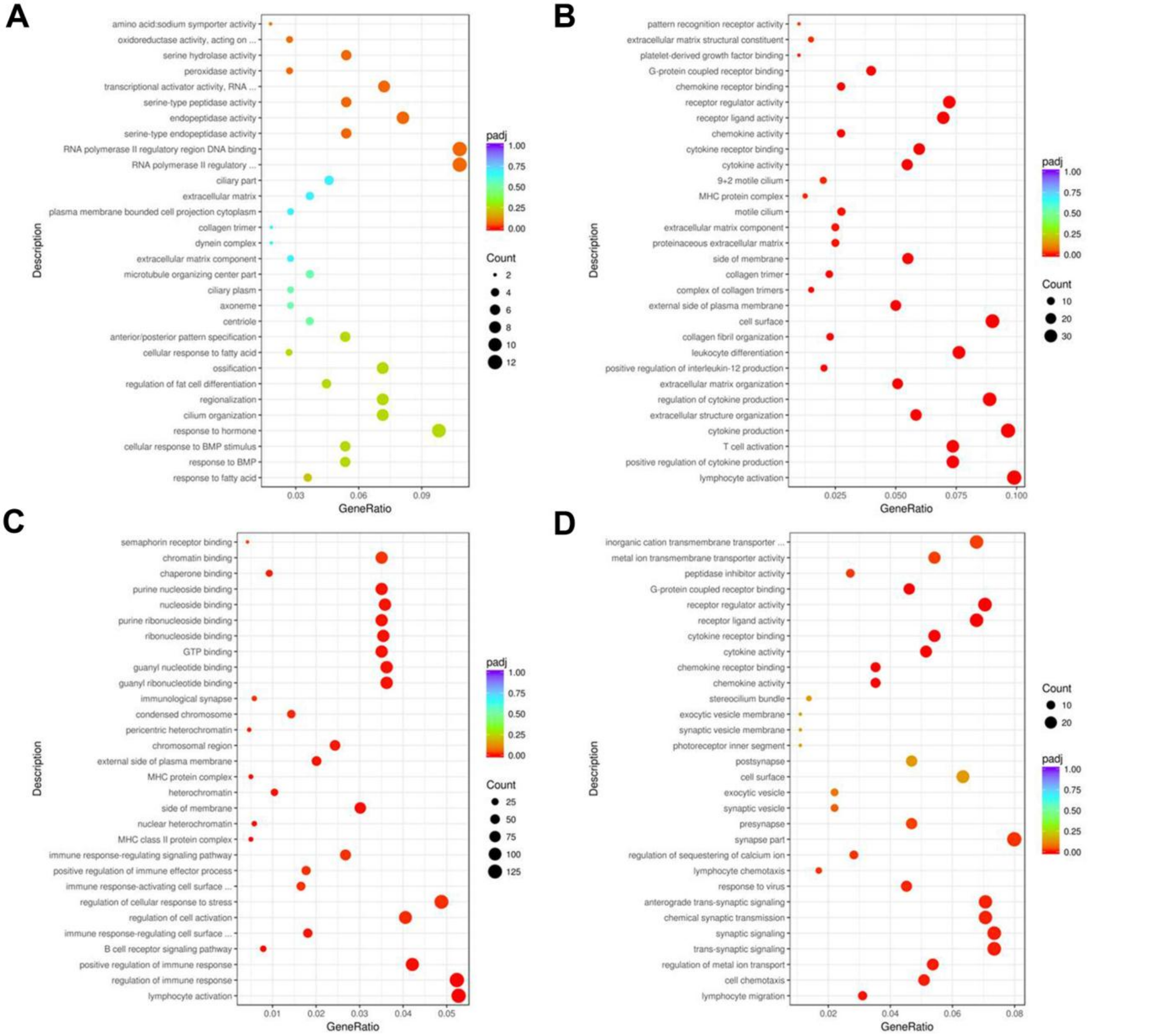

Supplementary Figure 3. Top gene ontology terms based on functional enrichment analysis of DEGs. The top 30 GO terms for (**A**) control vs. AR model, (**B**) control vs. AR model plus YJD, (**C**) AR model vs. AR model plus dexamethasone, and (**D**) AR model vs. AR model plus YJD groups.

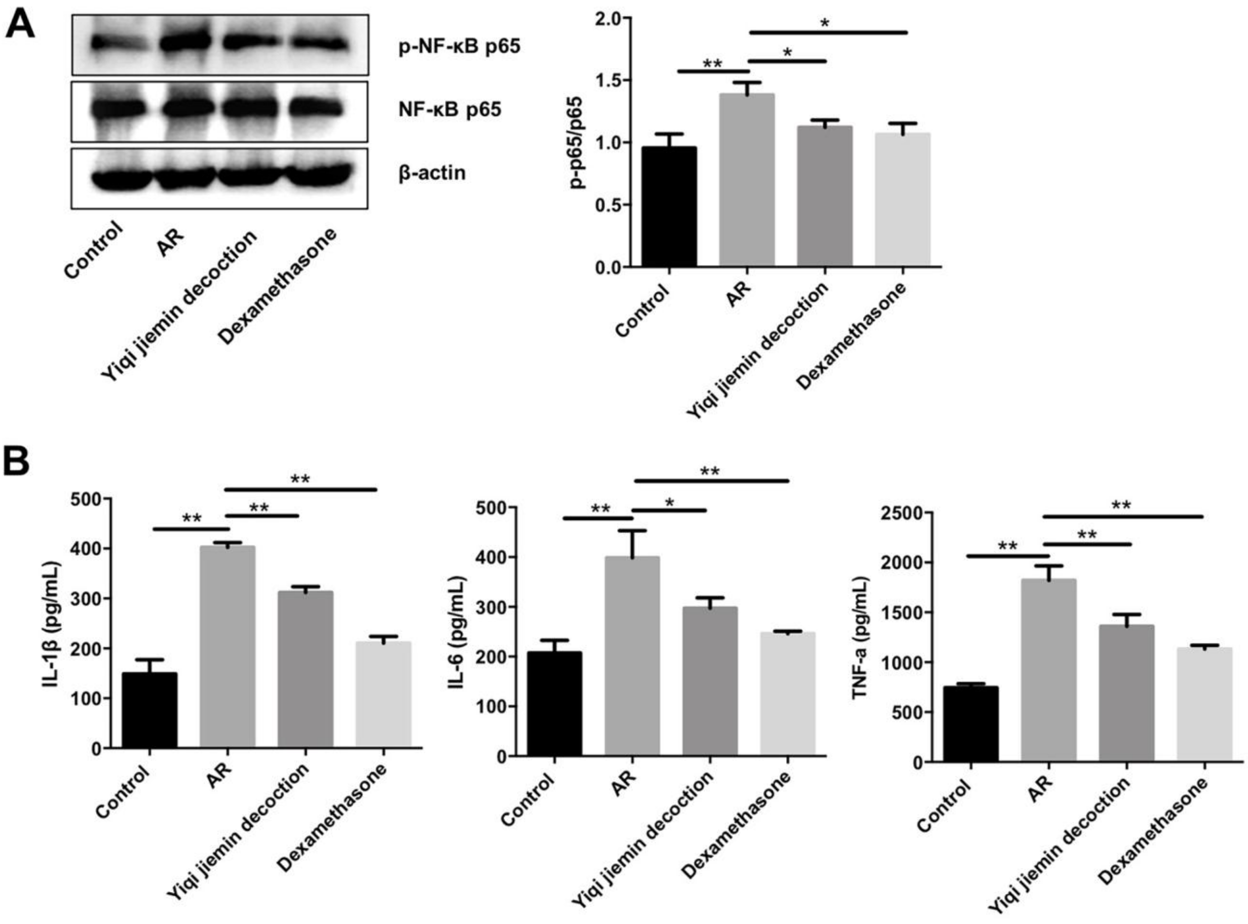

Supplementary Figure 4. YJD suppresses inflammation in a guinea pig model via inactivating NF-kappaB pathway. (**A**) Western blot analysis of the expressions of p-NF-κB and NF-κB in the control, AR model, AR model plus YJD, and AR model plus dexamethasone groups. (**B**) ELISA assay results show the serum levels of IL-6, TNF-α and IL-1β in the control, AR model, AR model plus YJD, and AR model plus dexamethasone groups. *P < 0.05; **P < 0.01; one-way ANOVA.
